# Gut microbiota, liver disease, and perioperative anesthesia: interactions, risks, and therapeutic opportunities

**DOI:** 10.3389/fcimb.2025.1759076

**Published:** 2026-01-22

**Authors:** Lei Shi, Ye Yu, Zihan Ma, Weiyi Jiang

**Affiliations:** Department of Anesthesiology, The Affiliated People’s Hospital of Ningbo University, Ningbo, China

**Keywords:** anesthesia, gut microbiome, gut-liver axis, liver disease, perioperative care

## Abstract

Liver disease is increasingly common worldwide and poses significant challenges during anesthesia and surgery. Growing evidence demonstrates that the gut microbiome plays an essential role in hepatic inflammation, metabolic imbalance, immune dysfunction, and the progression of conditions such as metabolic associated steatotic liver disease, alcohol related liver injury, and cirrhosis. This review summarizes the concept of the gut-liver-anesthesia axis, which describes how disturbances in the intestinal microbiome shape perioperative risk. Importantly, this framework conceptualizes the gut-liver-anesthesia axis as a unified perioperative risk model, integrating microbial dysbiosis, hepatic vulnerability, and anesthetic exposure into a single pathophysiological continuum. Patients with advanced liver disease frequently exhibit reduced microbial diversity, impaired intestinal barrier function, disordered bile acid signaling, and heightened systemic inflammation. These alterations increase susceptibility to infection, kidney injury, hemodynamic instability, and neurocognitive complications including hepatic encephalopathy and postoperative delirium. Anesthetic agents can further disrupt the gut ecosystem by weakening mucosal integrity and facilitating bacterial translocation, while the microbiome itself influences drug metabolism and clearance, leading to unpredictable anesthetic responses. Understanding this bidirectional interaction highlights opportunities for microbiome focused perioperative strategies. Approaches such as probiotic based preparation, opioid sparing anesthesia, regional techniques, early enteral feeding, and targeted microbial restoration may improve postoperative outcomes in patients with liver disease.

## 1 Introduction

The global epidemiological landscape of chronic liver disease is undergoing a dramatic transformation that poses unprecedented challenges to perioperative medicine. While viral hepatitis has historically been the predominant etiology, the contemporary burden is increasingly driven by metabolic dysfunction-associated steatotic liver disease (MASLD) and alcohol-associated liver disease (ALD) (Younossi et al., 2024). Recent data indicate that MASLD has emerged as the fastest-growing cause of liver-related mortality worldwide, paralleling the rising prevalence of obesity and type 2 diabetes (Estes et al., 2018). Projections suggest that by 2040, the prevalence of MASLD in the adult population could exceed 55%, inevitably resulting in a surge of patients with compromised hepatic function presenting for non-hepatic surgical interventions (Le et al., 2022). Concurrently, ALD remains a persistent public health crisis, particularly in the Americas where incidence rates of alcohol-associated cirrhosis continue to climb (Younossi et al., 2023). Collectively, the expanding global burden of chronic liver disease is expected to translate into a rising incidence of anesthesia-related perioperative complications.

Patients with liver disease face a markedly elevated risk of perioperative morbidity and mortality (Jadaun and Saigal, 2022; Xiang et al., 2023). Individuals with decompensated cirrhosis carry a mortality risk 2 to 10 times higher than those without hepatic impairment, with outcomes strongly correlated with the severity of liver dysfunction (Mahmud et al., 2021; Wu et al., 2024). Beyond mortality, these patients are uniquely susceptible to a distinct triad of complications comprising infection, renal failure, and neuropsychiatric decline (O’leary et al., 2018). Surgical site infections and sepsis are frequent due to cirrhosis-associated immune dysfunction, while postoperative delirium affects a significant proportion of liver transplant recipients, severely impairing long-term cognitive recovery. Historically, anesthetic care has focused on preserving hepatic perfusion and managing altered drug pharmacokinetics (Gelman, 1987; Sharma et al., 2022; Zhang et al., 2022). However, this conventional approach overlooks a critical driver of hepatic pathology: the gut microbiota. The gut-liver axis, a bidirectional pathway connecting the intestine and the liver, is now recognized as a central regulator of hepatic health and systemic immunity (Albillos et al., 2020). In liver disease, this axis is profoundly disrupted by dysbiosis and intestinal barrier dysfunction, facilitating the translocation of bacteria and endotoxins into the portal circulation (Chassaing et al., 2017).

This review posits that the gut-liver axis represents a critical, yet underappreciated, theater of action in the perioperative period. We introduce the concept of the gut-liver-anesthesia axis, a framework describing how anesthetic agents and surgical stress exacerbate pre-existing gut dysbiosis, which in turn alters anesthetic pharmacology and perioperative outcomes (Zhang et al., 2025). Unlike the traditional gut-liver axis, which primarily focuses on chronic metabolic and inflammatory crosstalk, the gut-liver-anesthesia axis explicitly incorporates perioperative exposures as dynamic modifiers of microbial and hepatic vulnerability. Emerging evidence suggests that volatile anesthetics can directly impair the intestinal barrier, acting as a second hit to the vulnerable liver (Zhao et al., 2024). Conversely, the gut microbiota regulates the expression of key hepatic drug-metabolizing enzymes, thereby influencing the efficacy and toxicity of perioperative medications (Collins and Patterson, 2020). The objective of this report is to synthesize current evidence on these interactions, elucidating how the microbiome influences anesthetic response and exploring microbiome-targeted strategies to improve surgical outcomes in patients with liver disease.

## 2 The gut-liver axis in liver disease: mechanisms relevant to anesthesia

The anatomical and functional integration of the liver and intestine creates a system where pathology in one organ inevitably impacts the other. The gut-liver axis serves as the conduit for this crosstalk, exposing the liver to gut-derived factors via the portal vein and the intestine to liver-derived bile acids and antibodies (Rai et al., 2015). In the context of liver disease, this axis transforms from a homeostatic regulator into a driver of systemic inflammation and metabolic derangement.

### 2.1 Gut dysbiosis in liver disease

At the core of gut-liver axis dysfunction in liver disease lies a profound alteration in microbial composition and function, commonly referred to as gut dysbiosis. The transition from a healthy liver to decompensated cirrhosis is accompanied by a progressive and catastrophic shift in the gut ecosystem. This microbiome compositional dysbiosis is quantitatively characterized by a reduction in the cirrhosis dysbiosis ratio, which reflects an imbalance between beneficial autochthonous taxa and potentially pathogenic species (Bajaj et al., 2014). In a healthy state, families such as *Lachnospiraceae* and *Ruminococcaceae* maintain epithelial integrity through the production of short-chain fatty acids like butyrate (Khan et al., 2021). In advanced liver disease, these beneficial populations collapse, replaced by an overgrowth of *Proteobacteria*, specifically *Escherichia coli* and *Klebsiella pneumoniae*, which drive ammonia production and endotoxemia (Jasirwan et al., 2019). The specific nature of this dysbiosis varies by etiology. Alcohol-associated liver disease is characterized by an enrichment of ethanol-producing bacteria that generate endogenous alcohol and acetaldehyde, subjecting the liver to continuous oxidative stress even in the absence of dietary alcohol intake (Park et al., 2021). Similarly, in MASLD, dysbiosis alters choline metabolism to produce trimethylamine, a precursor to the pro-inflammatory metabolite trimethylamine N-oxide, which is linked to cardiovascular risk and hepatic inflammation (Violi et al., 2023).

### 2.2 Intestinal barrier disruption

Beyond compositional changes in the gut microbiota, dysbiosis in liver disease directly compromises the structural and functional integrity of the intestinal barrier. A critical consequence of dysbiosis in liver disease is the systematic dismantling of the intestinal barrier, often referred to as leaky gut. This barrier failure is multifactorial, involving the disassembly of tight junction proteins such as zonula occludens-1 and occludin due to elevated levels of inflammatory cytokines and bacterial endotoxins (Pérez et al., 2019). In ALD, ethanol and its metabolites directly dissolve lipids and form protein adducts that further compromise epithelial integrity (Liu et al., 2020). Additionally, the depletion of mucin-regulating bacteria like *Akkermansia muciniphila* leads to a thinning of the protective mucus layer, allowing bacteria direct contact with the epithelium. The resulting increase in permeability permits the pathological translocation of viable bacteria and lipopolysaccharides from the intestinal lumen into the portal circulation (Giles et al., 2015). Under normal conditions, hepatic Kupffer cells clear these antigens; however, in liver disease, this clearance capacity is overwhelmed. The binding of lipopolysaccharides to Toll-like receptor 4 (TLR4) on hepatic stellate cells triggers a potent inflammatory cascade mediated by nuclear factor kappa B, releasing cytokines that prime the liver for acute decompensation during surgical stress (Chen et al., 2024).

### 2.3 Altered bile acid signaling

In addition to barrier dysfunction, dysbiosis profoundly reshapes bile acid metabolism and signaling, thereby linking microbial alterations to hepatic metabolic regulation. Bile acids function as potent metabolic signaling molecules that regulate both hepatic and intestinal physiology. Primary bile acids synthesized in the liver are modified by the gut microbiota into secondary bile acids, a process that is significantly altered in the setting of dysbiosis (Serbanescu et al., 2019). The resulting skewed bile acid pool fails to appropriately activate the farnesoid X receptor (FXR) in the distal ileum, leading to suppression of fibroblast growth factor 19 secretion (Liu et al., 2021). This disruption of the FXR-FGF19 axis results in unchecked bile acid synthesis and hepatic accumulation, exacerbating cholestatic liver injury. Crucially for the anesthesiologist, bile acid receptors also regulate the expression of key hepatic drug-metabolizing enzymes. The dysregulation of this signaling pathway in cirrhotic patients correlates with downregulated cytochrome P450 3A4 (CYP3A4) expression, providing a mechanistic explanation for the unpredictable pharmacokinetics and prolonged duration of action often observed with anesthetic drugs in this population (Huang et al., 2021).

### 2.4 Immune dysregulation

These combined microbial, barrier, and metabolic disturbances ultimately converge on the host immune system, resulting in a state of chronic immune dysregulation in liver disease. The gut microbiota acts as the primary educator of the host immune system, particularly influencing the balance of pro-inflammatory and anti-inflammatory cells. Specific commensal bacteria adhere to the intestinal epithelium and induce the differentiation of T helper 17 cells, which produce interleukin-17 (De Kock et al., 2013). In liver disease, this axis becomes maladaptive. While interleukin-17 is essential for mucosal defense, its chronic overproduction in response to dysbiosis drives systemic inflammation and neutrophil recruitment to the liver (Zhang and Roy, 2021). This pre-existing state of chronic immune activation means that patients with liver disease are primed to mount an exaggerated inflammatory response to surgical trauma. The heightened baseline inflammation increases the risk of developing systemic inflammatory response syndrome and remote organ dysfunction, such as acute lung injury, where the gut-lung axis facilitates the migration of activated neutrophils and inflammatory mediators (Tarantino et al., 2024).

## 3 How anesthetic agents perturb the gut microbiome and intestinal barrier

**Figure 1:**
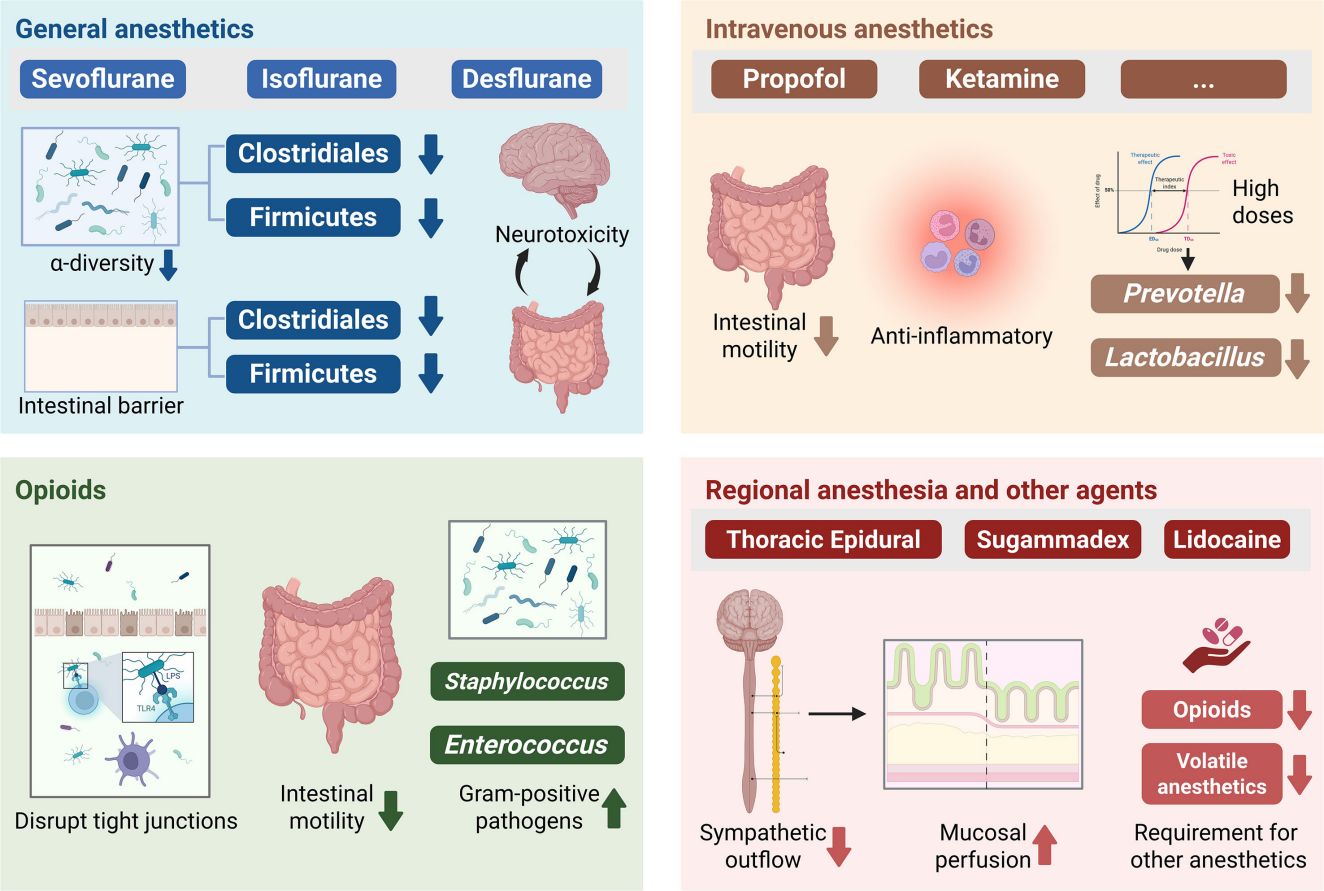
How anesthetic agents perturb the gut microbiome and intestinal barrier.

The administration of anesthesia is not a biologically neutral event but rather a significant ecological disturbance that can acutely reshape the gut microbiome. This section examines how different classes of anesthetic agents interact with the gut-liver axis, potentially exacerbating the underlying pathology in patients with liver disease (**Figure 1**, **Table 1**).

**Table 1:** Comparative effects of anesthetic agents on the gut microbiome and barrier function.

Anesthetic agent	Effect on microbiome composition	Impact on intestinal barrier	Mechanism of action	Clinical risk in liver disease
Sevoflurane	Decreased Diversity: Depletion of *Firmicutes*, *Clostridiales*; relative increase in *Proteobacteria*.	Impaired: Downregulation of ZO-1 and occludin; mucus layer thinning.	Direct toxicity; altered motility; immune modulation.	High: Promotes translocation; links to postoperative cognitive dysfunction.
Opioids	Severe Dysbiosis: Expansion of *Enterococcus*, *Staphylococcus*; reduction in *Lachnospiraceae*.	Severely Impaired: TLR4-mediated TJ disruption; stasis promotes SIBO.	Mu-opioid receptor activation on enteric neurons and epithelium.	Very High: Drives bacterial translocation, SBP, and HE precipitation.
Propofol	Neutral/Variable: Transient decrease in *Lactobacillus*; less perturbation than volatiles.	Modulated: Anti-inflammatory effects may blunt LPS toxicity.	Immune modulation (anti-inflammatory); hemodynamic changes.	Moderate: Preferred for HE management but hypotension risks gut ischemia.
Remimazolam	Under Investigation: May have milder impact on motility than propofol.	Preserved: Better hemodynamic stability supports perfusion.	GABA-A agonist; organ-independent metabolism.	Low/Moderate: Promising for hemodynamically unstable cirrhotics.
Sugammadex	Potential Prebiotic: Cyclodextrin structure may be fermented by microbiota.	Neutral: No direct barrier toxicity reported.	Encapsulation of steroidal NMBAs.	Low: Rapid reversal avoids residual paralysis and respiratory complications.
Lidocaine	Beneficial: Potential to reduce pathogen virulence; anti-inflammatory.	Protected: Reduces ischemia-reperfusion injury (debated efficacy in minimally invasive surgery).	Sodium channel blockade; reduction in sympathetic tone.	Low: Protective; key for opioid-sparing protocols.

### 3.1 General anesthetics: volatile agents

Volatile anesthetics, including sevoflurane, isoflurane, and desflurane, are cornerstones of general anesthesia, yet they exert profound and often deleterious effects on the gut ecosystem. Animal models have consistently demonstrated that exposure to these agents precipitates a rapid reduction in bacterial alpha-diversity, specifically depleting beneficial orders such as *Clostridiales* and the *Firmicutes* phylum (Thomas et al., 2022). This loss of diversity diminishes colonization resistance, creating a niche for potential pathogens to proliferate. Beyond taxonomic shifts, volatile agents appear to have direct cytotoxic effects on the intestinal epithelium. Research in neonatal models has shown that repeated exposure to sevoflurane leads to significant damage to the mucus layer and disruption of tight junctions, characterized by the downregulation of occludin and zonula occludens-1 (Ananda et al., 2025). This barrier failure is clinically significant as it facilitates bacterial translocation and systemic inflammation. Furthermore, the impact of volatile anesthetics extends to the central nervous system via the gut-brain axis. Sevoflurane-induced dysbiosis has been causally linked to cognitive impairment, with fecal microbiota transplantation (FMT) from exposed donors sufficient to transfer memory deficits to germ-free recipients (Rao et al., 2024). This suggests that the neurotoxic effects of volatile anesthetics may be partially mediated by gut-derived metabolites and cytokines crossing the blood-brain barrier.

### 3.2 Intravenous anesthetics: propofol and ketamine

In contrast to volatile agents, intravenous anesthetics exhibit a more complex and potentially less damaging interaction profile with the gut microbiome. Propofol, commonly used for total intravenous anesthesia, profoundly inhibits intestinal motility during infusion, although gastrointestinal function typically recovers more rapidly than with volatile-based techniques (Wilson and Nicholson, 2017). While the lipid emulsion formulation of propofol can theoretically support bacterial growth, the drug itself possesses anti-inflammatory properties that may offer protection in the context of liver disease. Studies indicate that propofol can attenuate TLR4-mediated responses to endotoxins, potentially reducing the systemic impact of bacterial translocation (Harjumäki et al., 2021). However, high-dose propofol has been associated with changes in specific bacterial phyla, including a decrease in *Prevotella* and *Lactobacillus* (Center, 2023). Ketamine, an N-methyl-D-aspartate receptor antagonist, interacts with the gut-brain axis in a unique manner. It has been shown to exhibit anti-inflammatory effects in sepsis models, likely by interfering with nuclear factor kappa B activation (Zhang et al., 2023). Additionally, ketamine’s sympathomimetic properties help maintain splanchnic perfusion, which is critical for preserving intestinal barrier integrity in hemodynamically unstable patients with cirrhosis. Newer benzodiazepines such as remimazolam are being investigated for their microbiome profile, with early data suggesting a milder impact on motility compared to traditional agents, though robust microbiome sequencing data remains emerging (Zhang et al., 2023).

### 3.3 Opioids and the gut ecosystem

Opioids represent the most disruptive class of perioperative medications regarding the gut-liver axis, exerting deleterious effects through multiple mechanisms. The primary insult is the inhibition of peristalsis via mu-opioid receptors in the enteric nervous system, leading to opioid-induced bowel dysfunction (Giner Pérez et al., 2025). This stasis promotes small intestinal bacterial overgrowth, a condition already prevalent in cirrhosis that significantly increases the bacterial burden available for translocation. Beyond motility, opioids directly compromise the intestinal barrier. Morphine has been shown to disrupt tight junction organization via a TLR4-dependent mechanism, increasing permeability even in the absence of stasis (Velasco et al., 2024). Furthermore, both acute and chronic opioid exposure induce a specific dysbiotic signature characterized by the expansion of Gram-positive pathogens such as *Staphylococcus* and *Enterococcus* (Wu et al., 2025). This shift is particularly dangerous for patients with liver disease, as *Enterococcus* species are frequent causative agents of spontaneous bacterial peritonitis and bacteremia. The resulting dysbiosis and barrier failure can create a vicious cycle where systemic inflammation drives analgesic tolerance, necessitating higher opioid doses that further damage the gut (Yang et al., 2024).

### 3.4 Regional anesthesia and other agents

Regional anesthesia techniques, particularly thoracic epidural anesthesia, offer a distinct physiological advantage by mitigating the gut-damaging effects of surgical stress and systemic drugs. Epidural anesthesia blocks sympathetic outflow to the splanchnic vascular bed, which can improve mucosal perfusion and reduce the risk of ischemia-reperfusion injury during major abdominal surgery (Xu et al., 2025). This preservation of microcirculation is vital for maintaining barrier function. Furthermore, by providing potent analgesia, regional techniques significantly reduce the requirement for systemic opioids and volatile anesthetics, thereby minimizing the direct toxic exposure of the microbiome to these agents. Other perioperative drugs also interact with the microbiome; for instance, sugammadex, a cyclodextrin-based reversal agent, has been shown to encapsulate not only neuromuscular blockers but potentially interact with the bacterial cell wall or metabolites, although the clinical significance of this interaction on dysbiosis is still under investigation. (Ma et al., 2024) The use of lidocaine, particularly in intravenous infusions, has shown anti-inflammatory properties that may preserve gut barrier function and reduce postoperative ileus, further highlighting the protective potential of opioid-sparing strategies (Song et al., 2025).

## 4 How gut dysbiosis modifies anesthetic response and perioperative risk

The interaction between the patient and the anesthetic is reciprocal. Just as anesthesia affects the microbiome, the pre-existing dysbiosis in liver disease fundamentally alters the patient’s physiological and pharmacological response to anesthesia. This bidirectional relationship creates a unique microbiome-driven risk profile (**Table 2**).

**Table 2:** Evidence-based microbiome interventions for the liver surgical patients.

Intervention	Mechanism	Clinical outcome
Perioperative Synbiotics	Increases *Lactobacillus/Bifidobacterium*; reinforces TJs; reduces endotoxemia.	Reduces Infection: Lower Risk Ratio for postoperative infections in liver surgery. Shortens antibiotic use.
Opioid-Free Anesthesia	Prevents motility stasis; avoids TLR4-mediated barrier injury.	Hemodynamic Stability: Improved stability during hepatectomy; reduced vasopressor use.
Rifaximin Prophylaxis	Modulates flora to reduce ammonia-producers and endotoxin load.	Prevents HE: Reduces risk of overt HE episodes; reduces SBP recurrence.
Intravenous Lidocaine	Anti-inflammatory; sympathetic modulation; opioid-sparing.	Gut Protection: Mixed results; anti-inflammatory in open surgery but no gut benefit in minimally invasive.
Fecal Microbiota Transplant	Replaces dysbiotic flora; restores secondary bile acid metabolism.	Rescues Function: Significantly reduces HE recurrence; safe in decompensated cirrhosis.
Branched-Chain Amino Acids	Promotes protein synthesis; immune modulation.	Improves Recovery: Reduces postoperative infection and length of hospital stay.
Carbohydrate Loading	Reduces insulin resistance; provides substrate for gut bacteria.	Metabolic Control: Reduces perioperative insulin resistance in liver donors; safe.

### 4.1 The pharmacomicrobiomics of anesthesia

The gut microbiome contributes significantly to drug metabolism, a field known as pharmacomicrobiomics, which becomes critical when hepatic metabolic capacity is compromised. Gut bacteria express a vast array of enzymes, including beta-glucuronidases, sulfatases, and reductases, which can metabolize drugs before absorption or reactivate biliary-excreted metabolites. For instance, bacterial beta-glucuronidases can deconjugate morphine-6-glucuronide, allowing the active parent drug to be reabsorbed into the circulation. In dysbiosis, the alteration of bacterial populations renders this enterohepatic recycling unpredictable, potentially leading to opioid re-sedation and respiratory depression hours after administration (Macmillan, 2020). More profoundly, the gut microbiota regulates the expression of hepatic cytochrome P450 enzymes. Research in germ-free models has demonstrated that microbiota-derived signals are essential for the basal expression of CYP3A4, the enzyme responsible for metabolizing over half of all anesthetic drugs, including midazolam and fentanyl (Sharpton et al., 2022). In cirrhosis, the loss of CYP3A4-inducing bacteria or the suppression of nuclear receptors like FXR contributes to the reduced clearance of these agents. Similarly, the expression of CYP2E1, which metabolizes acetaminophen and volatile anesthetics, is modulated by microbial metabolites. In ALD, dysbiosis may induce CYP2E1 activity, paradoxically increasing the risk of hepatotoxicity from drugs metabolized by this pathway (Hong et al., 2022).

### 4.2 Hepatic encephalopathy and postoperative delirium

Hepatic encephalopathy (HE) and postoperative delirium are devastating neuropsychiatric complications that are intimately linked to the gut microbiome. The microbiome acts as the primary engine of HE by producing neurotoxins, most notably ammonia, via urease-positive bacteria such as *Klebsiella* and *Proteus*, which are enriched in the cirrhotic gut. Anesthesia and surgery exacerbate this burden by inducing constipation and catabolic stress, which increase ammonia production and retention (Kahn et al., 2020). Beyond ammonia, specific microbiome signatures have been identified as independent predictors of postoperative delirium. A relative abundance of *Parabacteroides distasonis* and a depletion of beneficial *Lachnospiraceae* have been linked to cognitive decline after surgery (Goff et al., 2023). The underlying mechanism involves a neuro-inflammatory priming effect: gut-derived endotoxins cross the compromised intestinal barrier and activate microglia in the brain. The stress of anesthesia and surgery then triggers an exaggerated release of neurotoxic cytokines from these primed cells, resulting in acute cognitive failure (Cheng et al., 2021). This gut-immune-brain pathway explains why even minor surgical procedures can precipitate severe encephalopathy or delirium in patients with liver disease.

### 4.3 Microbiome-driven systemic complications

The dysbiotic gut serves as a reservoir for pathogens and a driver of systemic organ failure. Bacterial translocation is the primary mechanism underlying spontaneous bacterial peritonitis and postoperative sepsis. In liver resection or transplantation, the manipulation of the bowel and interruption of portal blood flow can cause a massive influx of bacteria and endotoxins into the systemic circulation (Sun et al., 2014). Patients with a low cirrhosis dysbiosis ratio are at significantly higher risk for these infectious complications. Furthermore, the local microbiome plays a direct role in surgical healing. Collagenolytic bacteria, such as *Enterococcus faecalis*, can degrade collagen at anastomotic sites, leading to leakage and dehiscence (Shogan et al., 2015). In cirrhotic patients, who already suffer from impaired protein synthesis, the enrichment of these pathobionts dramatically increases the risk of anastomotic failure (Tsiaoussis et al., 2015). Additionally, the gut-kidney axis is mediated by endotoxemia; circulating lipopolysaccharides cause systemic vasodilation and renal vasoconstriction, precipitating hepatorenal syndrome (Tsuji et al., 2024). Perioperative worsening of barrier function can thus trigger acute kidney injury, a complication associated with high mortality in this population (Zarbock et al., 2018).

### 4.4 Anesthetic characteristics of specific liver disease phenotypes

Different etiologies of liver disease present unique anesthetic challenges driven by their specific microbial profiles. Patients with MASLD often exhibit a phenotype characterized by obesity and chronic low-grade inflammation. This systemic inflammation increases the requirement for anesthetic agents and elevates the risk of respiratory complications (Huth et al., 2011). In contrast, patients with ALD have profound dysbiosis with a high burden of ethanol-producing bacteria and severe barrier dysfunction, making them exceptionally prone to postoperative infections and wound healing complications (Meroni et al., 2019). For patients with advanced cirrhosis of any etiology, the combination of severe dysbiosis, high intestinal permeability, and altered drug metabolism creates a state of extreme sensitivity to anesthesia, requiring meticulous titration of drugs and vigilant monitoring of neurological status (Li et al., 2019).

## 5 Opportunities for microbiome-targeted perioperative management

**Figure 2:**
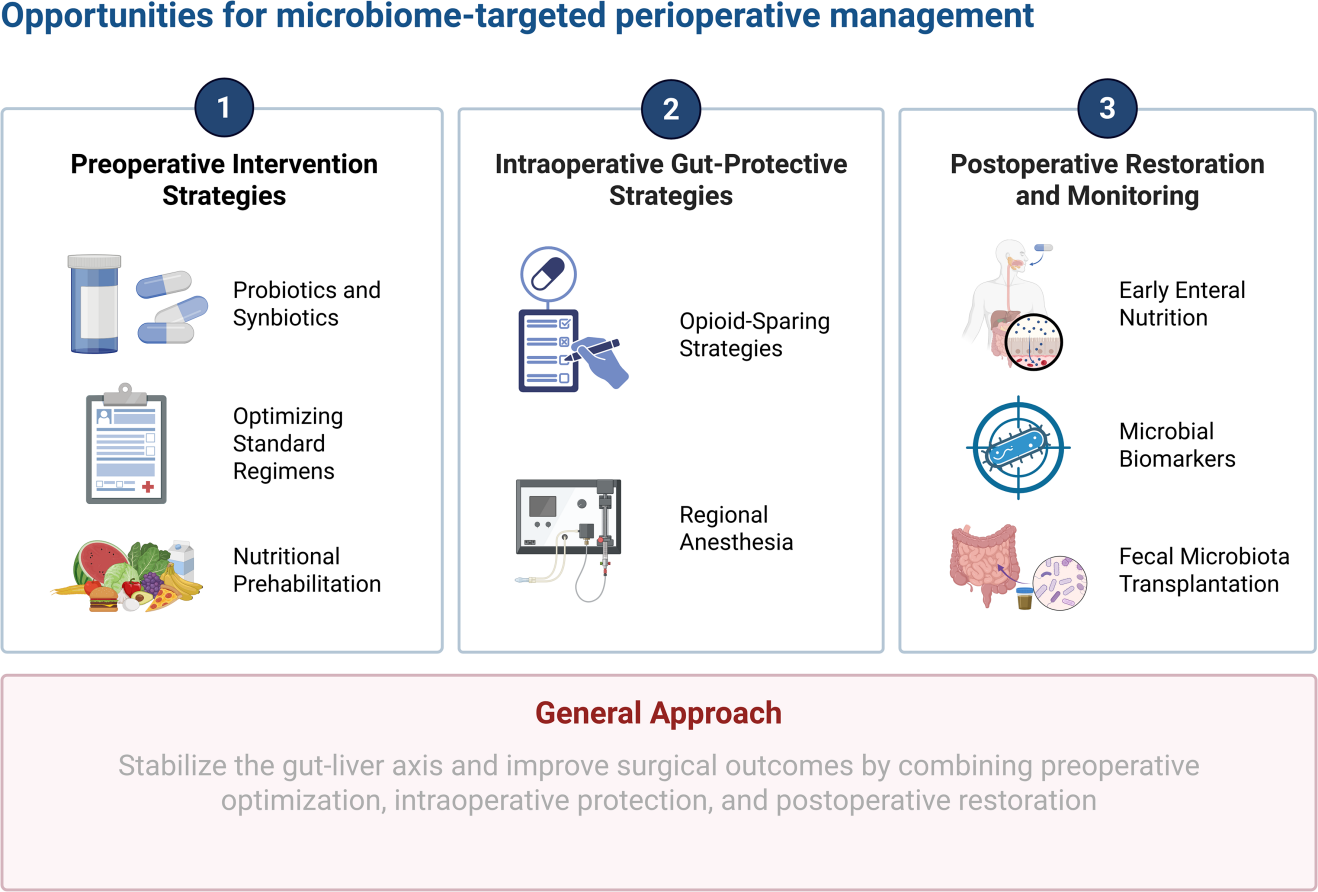
Opportunities for microbiome-targeted perioperative management.

The recognition of the gut microbiome as a modifiable risk factor opens new avenues for precision anesthesia. By targeting the gut ecosystem, clinicians can potentially stabilize the gut-liver axis and improve perioperative outcomes through a combination of preoperative optimization, intraoperative protection, and postoperative restoration (**Figure 2**).

### 5.1 Preoperative intervention strategies

The preoperative period offers a crucial window to fortify the gut-liver axis. Probiotics and synbiotics have shown substantial promise in this setting. Meta-analyses of randomized controlled trials indicate that perioperative administration of synbiotics, containing strains such as *Lactobacillus* and *Bifidobacterium*, significantly reduces the incidence of postoperative infections and shortens antibiotic therapy duration in patients undergoing liver transplantation and resection (Wu et al., 2025). These interventions work by reinforcing epithelial tight junctions, enhancing mucosal immunity, and competitively excluding pathogens. For patients with cirrhosis, particularly those with a history of encephalopathy, optimizing the standard prophylactic regimen of rifaximin and lactulose is essential. Rifaximin modulates the microbiota to reduce ammonia-producing species and endotoxin load, thereby lowering the risk of postoperative HE (Li et al., 2025). Nutritional prehabilitation also plays a vital role; enteral nutrition is superior to parenteral routes for maintaining barrier function, and supplementation with dietary fiber and branched-chain amino acids can improve microbial diversity and metabolic resilience (Wobith et al., 2024). Preoperative carbohydrate loading has also been shown to reduce perioperative insulin resistance and support functional liver recovery, potentially by providing substrate for beneficial gut bacteria (Wobith et al., 2024).

### 5.2 Intraoperative gut-protective strategies

Minimizing iatrogenic damage to the microbiome is the cornerstone of intraoperative management. Opioid-free or opioid-sparing anesthesia protocols are increasingly advocated to prevent the deleterious effects of opioids on gut motility and barrier function. The use of multimodal analgesia, incorporating intravenous lidocaine, ketamine, and dexmedetomidine, can drastically reduce opioid requirements. Intravenous lidocaine, in particular, has demonstrated anti-inflammatory properties and the ability to accelerate the return of bowel function, offering protection against ischemia-reperfusion injury (Fernández-Martínez et al., 2025). Regional anesthesia, such as thoracic epidural analgesia, is highly beneficial in open liver surgery. By blocking sympathetic outflow, it improves splanchnic perfusion and reduces the neuroendocrine stress response, potentially preserving gut barrier integrity (Sertcakacilar et al., 2022). Regarding general anesthetics, while definitive trials comparing total intravenous anesthesia versus volatile anesthesia on microbiome outcomes are ongoing, the use of propofol may be theoretically preferable in high-risk patients due to its lesser impact on barrier function compared to volatile agents (Ramirez and Gan, 2023).

### 5.3 Postoperative restoration and monitoring

Postoperative care must focus on the rapid restoration of the gut ecosystem. Early enteral feeding is the most potent stimulus for maintaining mucosal mass and barrier function; it is strongly recommended by enhanced recovery after surgery protocols to prevent bacterial translocation (Zheng et al., 2023). Future management may involve the use of microbial biomarkers for risk stratification. Signatures such as the 13-species microbial signature have been validated to predict the risk of hepatic decompensation and mortality, potentially allowing for the identification of high-risk patients who require intensive monitoring (Sharpton et al., 2022). FMT represents a frontier in postoperative care. While currently investigational in the perioperative setting, its efficacy in treating recurrent HE and severe alcoholic hepatitis suggests it could be used to rescue patients with severe postoperative dysbiosis or antibiotic-associated diarrhea (Tian et al., 2024). The concept of autologous fecal transplant offers a personalized approach to restoring microbial diversity after the insult of surgery and antibiotics.

## 6 Conclusion

The gut-liver-anesthesia axis represents a clinically actionable framework that fundamentally redefines the understanding of perioperative risk in patients with liver disease. The evidence synthesized in this review demonstrates that the gut microbiome is not merely a passive bystander but an active determinant of hepatic physiology and surgical outcomes. The dysbiotic gut, characterized by a loss of diversity, barrier failure, and altered metabolic signaling, creates a primed environment that amplifies the stress of surgery and anesthesia, predisposing patients to complications ranging from infection to encephalopathy. Crucially, the anesthesiologist’s pharmacological tools are double-edged swords; while necessary for surgery, agents like opioids and volatile anesthetics can act as second hits to this fragile system. Conversely, the patient’s unique microbial signature dictates the pharmacokinetic reality of the operating room.

As we move towards the next decade, the integration of microbiome stewardship into perioperative care, through prehabilitation of the gut, microbiome-sparing anesthetic techniques, and targeted restorative therapies, will be pivotal in improving the safety and survival of the growing population of patients with liver disease. In the near future, microbiome-based biomarkers and risk stratification tools may be incorporated into perioperative pathways to identify vulnerable patients and personalize anesthetic and surgical strategies.
